# Small leucine zipper protein functions as a negative regulator of estrogen receptor α in breast cancer

**DOI:** 10.1371/journal.pone.0180197

**Published:** 2017-06-29

**Authors:** Juyeon Jeong, Sodam Park, Hyoung-Tae An, Minsoo Kang, Jesang Ko

**Affiliations:** Division of Life Sciences, Korea University, Seoul, South Korea; Roswell Park Cancer Institute, UNITED STATES

## Abstract

The nuclear transcription factor estrogen receptor α (ERα) plays a critical role in breast cancer progression. ERα acts as an important growth stimulatory protein in breast cancer and the expression level of ERα is tightly related to the prognosis and treatment of patients. Small leucine zipper protein (sLZIP) functions as a transcriptional cofactor by binding to various nuclear receptors, including glucocorticoid receptor, androgen receptor, and peroxisome proliferator-activated receptor γ. However, the role of sLZIP in the regulation of ERα and its involvement in breast cancer progression is unknown. We found that sLZIP binds to ERα and represses the transcriptional activity of ERα in ERα-positive breast cancer cells. sLZIP also suppressed the expression of ERα target genes. sLZIP disrupted the binding of ERα to the estrogen response element of the target gene promoter, resulting in suppression of cell proliferation. sLZIP is a novel co-repressor of ERα, and plays a negative role in ERα-mediated cell proliferation in breast cancer.

## Introduction

Estrogens play critical roles in the growth and development of human breast epithelium [1]. The biological functions of estrogens are mediated by the estrogen receptor (ER) that belongs to the superfamily of nuclear hormone receptors [2]. Two ER isoforms, α and β, play important roles in the development and progression of estrogen-dependent cancers, including breast, ovarian, and cervical cancers [3, 4]. Since ERα is an important growth stimulatory transcription factor in breast cancer cells, regulation of ERα transcriptional activity is significant for breast cancer progression. ERα consists of three functional domains, which include a ligand-independent activation function (AF-1) domain, a highly conserved DNA-binding domain (DBD), and a ligand-binding domain (LBD) containing a dimerization and a ligand-dependent activation function (AF-2) domain [5–7].

In the classical model, binding of estrogen to ERα induces dissociation from heat shock proteins and ERα undergoes conformational changes, dimerization and translocation to the nucleus. [7, 8]. Activated nuclear ERα binds to the estrogen response element (ERE) in the promoters of estrogen-regulated genes, including pS2 and cyclin D1 [9, 10]. The transcriptional activity of ERα is enhanced by interaction with coactivators, including nuclear receptor coactivator 1 (NCoA1 or SRC1), NCoA2 (TIF2) and NCoA3 (AIB1, TRAM1, RAC3, or ACTR) to the AF-2 domain of ERα [8]. The protein complex enhances ERα-mediated transcription through multiple mechanisms such as recruitment of histone acetyltransferases (HATs), that give greater chromatin accessibility to the target gene promoter region [11]. Alternatively, corepressor proteins, including nuclear receptor corepressor 1 (NCoR1) and NCoR2, reduce ERα-induced transcription via recruitment of the histone deacetylase (HDAC) complex [12, 13].

An isoform of human leucine zipper protein (LZIP), known as small LZIP (sLZIP), consists of 354 amino acids, lacking a putative transmembrane domain (residues 229–245) of LZIP [14]. N-terminal of sLZIP contains a potent transcriptional activation domain composed of two LxxLL motifs [14]. LxxLL motifs are found in a number of transcriptional cofactors and mediate interaction with the nuclear hormone receptors [15]. sLZIP is localized in the nucleus, and functions as a transcriptional cofactor of various nuclear receptors, including glucocorticoid receptor (GR), androgen receptor (AR) and peroxisome proliferator-activated receptor γ_2_ (PPAR*γ*_2_) [14, 16, 17].

In this study, we characterize the role of sLZIP as a transcriptional corepressor of ERα in breast cancer cells. sLZIP physically interacts with ERα and suppresses binding of the ERα to ERE in response to estrogen, resulting in reduction of ERα target gene expression. sLZIP is a novel coregulator of ERα, and inhibits ERα-mediated estrogen signaling, leading to suppression of cell proliferation in breast cancer.

## Materials and methods

### Materials

Dulbecco’s Modified Eagle’s Medium (DMEM), RPMI 1640, penicillin, and streptomycin were purchased from Invitrogen (Carlsbad, CA, USA). Fetal bovine serum (FBS) was obtained from HyClone Laboratory (Logan, UT, USA). 17β-estradiol (E2) and charcoal were purchased from Sigma (St. Louis, MO, USA). Antibodies for ERα,pS2, cyclin D1, glutathione S-transferases (GST), Flag, and β-actin were obtained from Santa Cruz Biotechnology (Santa Cruz, CA, USA).

### Cell culture and transfection

MCF7 cells were maintained in DMEM supplemented with 10% FBS and penicillin (100 U/ml)/streptomycin (100 μg/ml). T47D and MDA-MB-231 cells were maintained in RPMI 1640 supplemented with 10% FBS and penicillin (100 U/ml)/streptomycin (100 μg/ml). For transient transfection, plasmids were transfected into cells using transfection reagent E-fection (Lugen Science, Gyeonggi, Korea), according to the manufacturer’s protocol.

### Luciferase reporter assay

Luciferase activity assay was performed using Dual-Luciferase Reporter Assay system (Promega, E1910). Cells were transfected with recombinant pGL4.21-ERE plasmid vector (Promega, E676A) and pRL-CMV Renilla plasmid vector (Promega, E2241). pRL-CMV Renilla plasmid vector was used to normalize the promoter luciferase activity. Cells were washed with cold-PBS and lysed with cell lysis buffer. Luciferase activity was recorded in Luminometer 20/20^n^ (Tuner Biosystems, Sunnyvale, CA, USA) according to the manufacturer’s protocol.

### cDNA plasmids and siRNAs

The wild-type sLZIP, N-terminal sLZIP (1–220), C-terminal sLZIP (221–354) and CC-terminal sLZIP (296–354) constructs were generated by a PCR [14]. All point mutations in two LxxLL motifs of sLZIP were generated by a QuikChange site-directed mutagenesis kit (Stratagene, Santa Clara, CA, USA). The 21-nucleotide sequences for the *siRNA* targeting sLZIP were 5′-CGUCGUUGAACAUUCUCAGdTdT-3′ (sense) and 5′-CUGAGAAUGUUCAACGACGdTdT-3′ (antisense). All *siRNA*s were synthesized from Bioneer (Daejeon, South Korea).

### Western blotting

Cells were washed twice with 1× PBS and cell extracts were prepared using RIPA buffer (1× PBS, 1% NP40, 0.5% sodium deoxycholate, 0.1% SDS, 0.5 mM PMSF, and protease inhibitors). Lysate proteins were resolved by 10% SDS-PAGE and transferred to nitrocellulose membranes (Whatman, GE healthcare, UK). The membranes were incubated with TBS buffer containing 0.1% Tween 20 and 5% skim milk, and exposed to the desired primary antibody. After treatment with a proper secondary antibody, the immunoreactive bands were visualized using standard ECL method (Pierce**,** Rockford**,** IL).

### Semi-quantitative RT-PCR

Total RNA was isolated using Trizol (Invitrogen) according to the manufacturer’s protocol. Approximately 2 μg of total RNA was used to prepare cDNA using a Superscript First Strand cDNA Synthesis Kit (Bioneer, Daejeon, South Korea). PCR was performed using specific primers (*sLZIP* sense 5′-ATGGAGCTGGAATTGGATGC-3′; antisense 5′-CTAGCCTGAGTATCTGTCCT-3′, *pS2* sense 5′-TGCTGTTTCGACGACACCGTT-3′; antisense 5′-AGGCAGATCCCTGCAGAAGT-3′, *GAPDH* sense 5′-CACCACCATGGAGAAGGCTGG-3′; antisense 5′-TTGTCATGGATGACCTTGGCCAGG-3′). *GAPDH* was used as an internal control. The PCR products were electrophoresed on a 1.5% (w/v) agarose gel in 1× Tris-acetate-EDTA (TAE) buffer, and stained with ethidium bromide solution. The intensity of each band amplified by RT-PCR was analyzed using ImageJ 1.46r (Wayne Rasband National Institutes of Health), and normalized to that of *GAPDH* mRNA in corresponding samples. Each experiment was performed in three experimental replicates, having three technical replicates within each experiment.

### Immunoprecipitation and GST pull-down assays

Immunoprecipitation assay was performed using MCF7 cells transfected with plasmids. Whole cell lysates were incubated overnight with 20 μl of protein A/G PLUS agarose (Santa Cruz) or glutathione Sepharose 4B bead slurry (GE Healthcare), at 4°C. Bound proteins were separated by SDS-PAGE, transferred to nitrocellulose membranes, and subjected to Western blot analysis using appropriate antibodies (Santa Cruz Biotechnology).

### Chromatin immunoprecipitation

MCF7 cells were grown in 100 mm plates. Confluent cultures were shifted to charcoal stripped media for 24 h and treated with or without 100 nM E2 for 24 h. Following treatment, cells were washed twice with PBS and cross-linked with 1% formaldehyde at 37°C for 10 min. Cells were then washed twice with PBS at 4°C, resuspended in lysis buffer (1% SDS, 10 mM EDTA, 50 mM Tris-HCl, pH 8.1), and left on ice for 10 min. Cells were sonicated four times for 10 s at 30% of maximal power (Fisher Sonic Dismembrator), and collected by centrifugation. The supernatants were collected and diluted in 1 ml of IP buffer (0.01% SDS, 1.1% Triton X-100, 1.2 mM EDTA, 16.7mM Tris-HCl pH 8.1, 16.7 mM NaCl) at 4°C. The chromatin was immunoprecipitated for 12 h using specific antibodies and anti-rabbit IgG as an internal control. Each sample was amplified from the prepared DNA using pS2 promoter primers flanking ERE-containing pS2 promoter fragment: sense 5′-GATTACAGCGTGAGCCACTG-3′ and antisense 5′-TGGTCAAGCTACATGGAAGG-3′.

### Cell proliferation assay

Viability of breast cancer cells was evaluated by the cell proliferation and colony forming assays. Cells were seeded in a 96 well plates at a density of 3 × 10^3^ cells/well. This was followed by treatment with or without 100 nM E2 for indicated time periods, in charcoal stripped media. Cell proliferation assay was performed using EZ-cytox (DoGenBio, Gyeonggi, Korea) according to the manufacturer’s protocol. For the colony forming assay, cells were seeded in 12 well plates at a density of 5 × 10^3^ cells/well. Cells were then treated with or without 100 nM E2. After 6 days, colonies were fixed with 4% paraformaldehyde for 30 min at room temperature and stained with 0.05% crystal violet solution.

### Statistical analysis

Data are presented as mean ± S.E.M. Statistical evaluation was performed with GraphPad Prism Software 5 (GraphPad Software, San Diego, CA, USA). Two-tailed *t*-test *p* values of 0.05 or less were considered as statistically significant.

## Results

### sLZIP down-regulates estrogen-responsive ERα transcriptional activity in ERα-positive breast cancer cells

**Fig 1:**
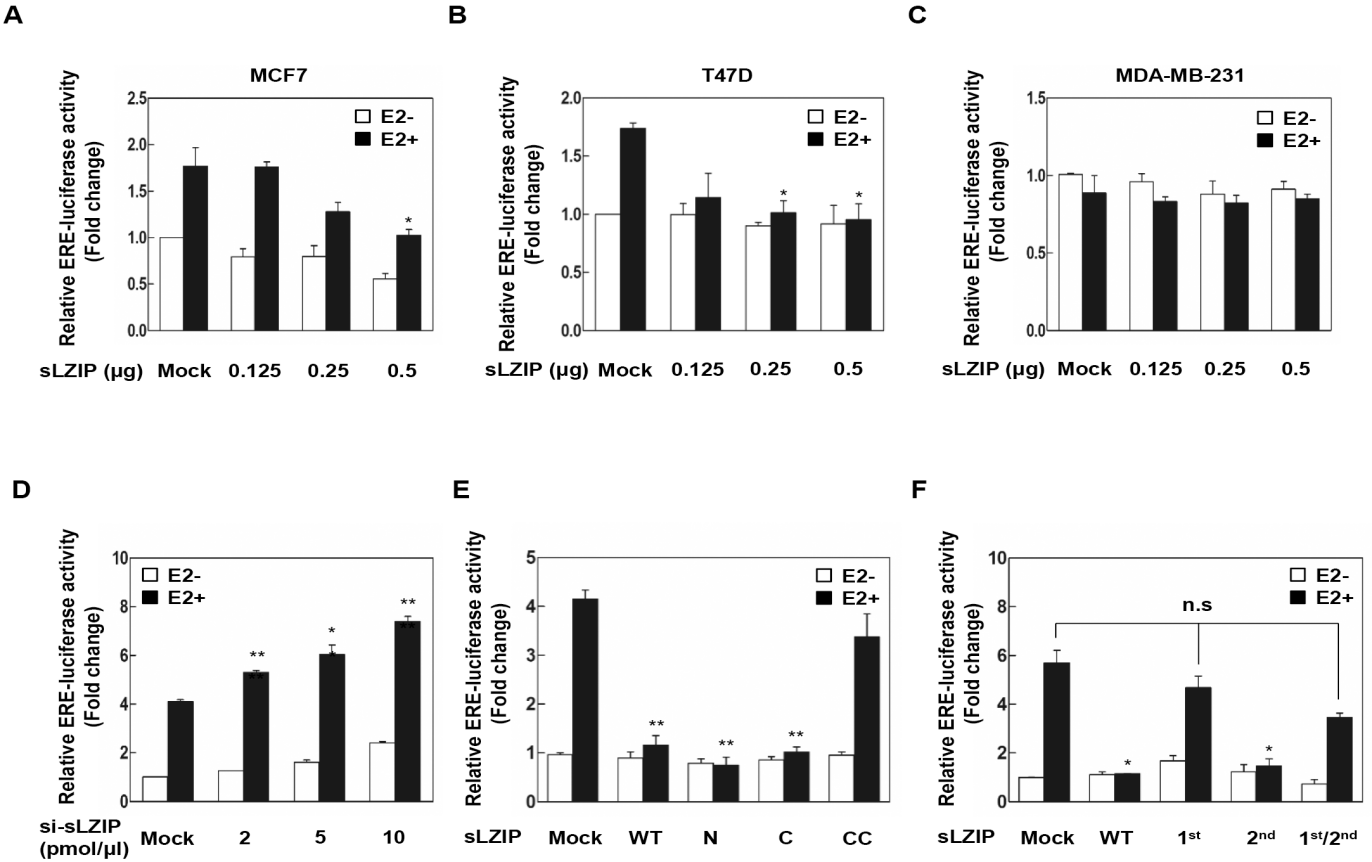
sLZIP down-regulates estrogen-responsive ERα transcriptional activity in ERα-positive breast cancer cells. (A-C) MCF7, T47D, and MDA-MB-231 cells were transfected with the ERE-Luc construct (0.5 μg), Renilla encoded vector (50 ng), and indicated amounts of GST-sLZIP. (D) MCF7 cells were transfected with the ERE-Luc construct (0.5 μg), Renilla encoded vector (50 ng), and indicated amounts of *si-sLZIP*. (E) MCF7 cells were transfected with the ERE-Luc construct (0.5 μg), Renilla encoded vector (50 ng), and sLZIP deletion mutants (0.5 μg), including N-terminal sLZIP (1–220), C-terminal sLZIP (221–354) and CC-terminal sLZIP (296–354). (F) MCF7 cells were transfected with the ERE-Luc construct (0.5 μg), Renilla encoded vector (50 ng), and two sLZIP-LxxLL mutants (0.5 μg). Cells were treated with or without 100 nM E2 for 12 h in charcoal stripped media. ERE luciferase activity was determined at 48 h post transfection. The experiments were performed in triplicate. Data are expressed as the mean ± S.E.M. and are presented as the relative luciferase activity. All *p* values were obtained using unpaired two-tailed *t*-test. *p < 0.05, **p < 0.01.

Since sLZIP contains two LxxLL nuclear receptor recognition motifs, we investigated whether sLZIP is involved in ERα-mediated transcription in breast cancer cells. We examined the effect of sLZIP on the transcriptional activity of ERα. ERα-positive MCF7 and T47D breast cancer cells were transiently transfected with GST-sLZIP and the ERE-luciferase (Luc) reporter gene. Cells were treated with E2 in charcoal stripped media, and luciferase activity was determined. E2 stimulated the transcriptional activity of endogenous ERα approximately 1.8-fold; however, sLZIP decreased ERα transcriptional activity in a dose-dependent manner in both cell lines (Fig 1A and 1B). sLZIP did not affect ERα transcriptional activity in ERα-negative MDA-MB-231 cells (Fig 1C). To investigate the role of endogenous sLZIP in regulation of ERα transcriptional activity, MCF7 cells were transfected with siRNA against sLZIP (*si-sLZIP*) and scrambled (*sc*) siRNA as a control. *si-sLZIP* increased ERα transcriptional activity in a dose-dependent manner upon E2 treatment (Fig 1D). To further investigate which domain of sLZIP is required for regulation of ERα transcriptional activity, we used deletion mutants of sLZIP, including N-terminal sLZIP (1–220), C-terminal sLZIP (221–354), and CC-terminal sLZIP (296–354). sLZIP N (1–220) and sLZIP C (221–354) inhibited ERα transactivation, whereas sLZIP CC (296–354) did not affect ERα transcriptional activity (Fig 1E). These results indicate that both N-terminal and C-terminal domains of sLZIP can inhibit ERα transcriptional activity. We next examined whether the two LxxLL motifs of sLZIP affect ERα transcriptional activity. We generated sLZIP-LxxLL mutants where Leu 16 and Leu 17 within the first LxxLL motif, and/or Leu 57 and Leu 58 within the second LxxLL motif were substituted with Ala. Luciferase assay was performed in MCF7 cells. The first sLZIP-LxxLL mutant abrogated the negative regulation of sLZIP for the E2-dependent transcriptional activity of ERα (Fig 1F). These results indicate that sLZIP negatively regulates the E2-dependent transcriptional activity of ERα, and the first LxxLL motif of sLZIP is required for regulation of ERα transcriptional activity.

### sLZIP represses expression of the estrogen-responsive genes

**Fig 2:**
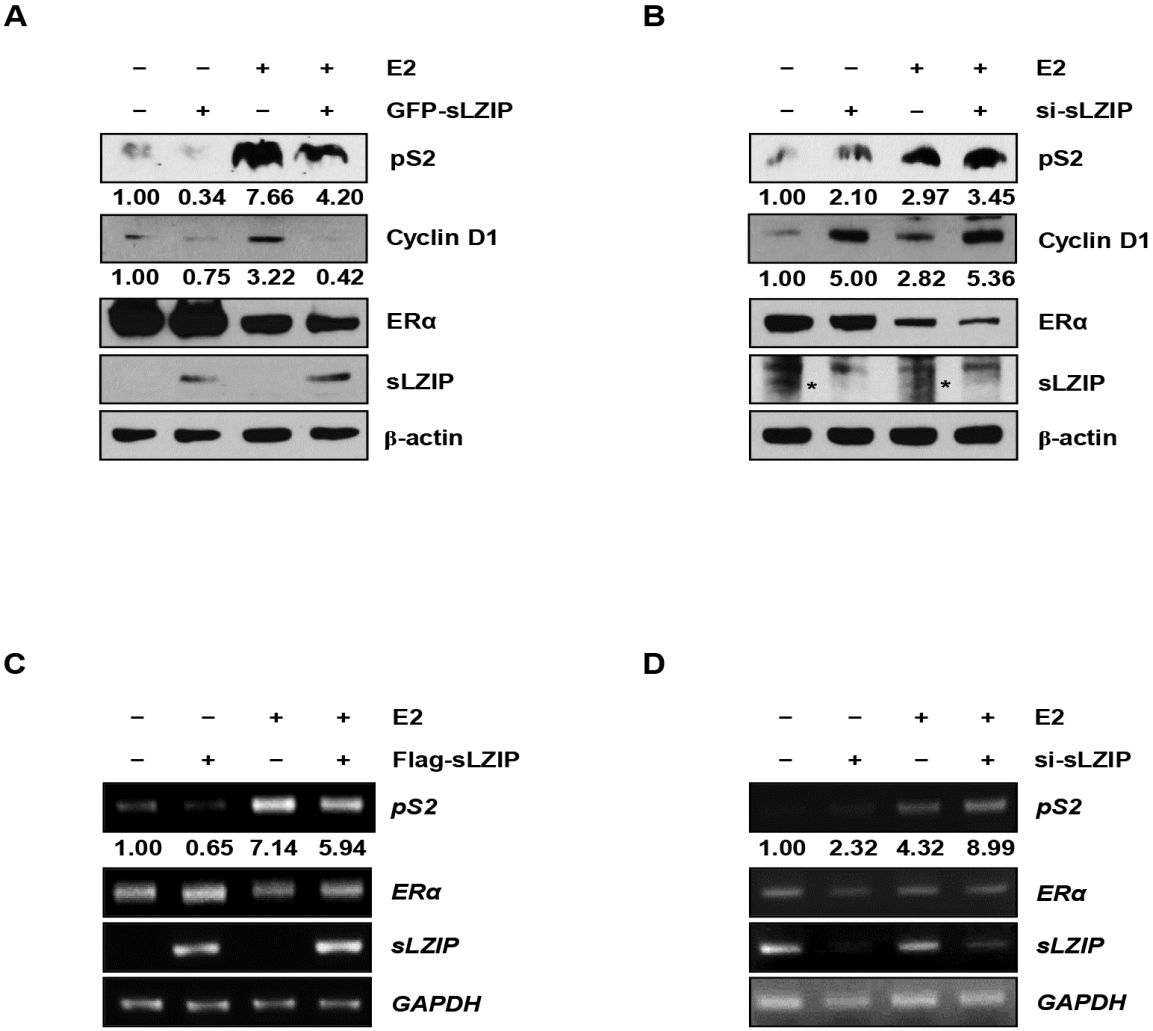
sLZIP represses expression of the estrogen-responsive genes. (A) MCF7 cells were transfected with GFP-mock or GFP-sLZIP (1 μg). (B) MCF7 cells were transfected with scrambled *siRNA* or *si-sLZIP* (100 pmol/μl). Cells were treated with or without 100 nM E2 for 24 h in charcoal stripped media. Total cell lysates were immunoblotted against anti-GFP, anti-ERα, anti-pS2, and anti-cyclin D1 antibodies. β-actin was used as an internal control. (C) MCF7 cells were transfected with Flag-mock or Flag-sLZIP (1 μg). (D) MCF7 cells were transfected with scrambled *siRNA* or *si-sLZIP* (100 pmol/μl). Cells were stimulated with or without 100 nM E2 for 24 h. Total RNA extracts were prepared and subjected to semi-qRT-PCR. GAPDH was used as an internal control.

We next examined the effect of sLZIP on expression of ERα target genes pS2 and cyclin D1. sLZIP did not influence the protein expression of ERα; however, sLZIP decreased the expression levels of both pS2 and cyclin D1, compared to a control (Fig 2A). We also investigated the effect of endogenous sLZIP on ERα target gene expression using *si-sLZIP*. sLZIP knockdown did not affect ERα expression; however, the expression levels of both pS2 and cyclin D1 were increased in cells transfected with *si-sLZIP*, compared to a control (Fig 2B). We examined the effect of sLZIP on the mRNA level of ERα target genes using semi-quantitative RT-PCR. sLZIP decreased the mRNA expression of pS2 (Fig 2C). sLZIP knockdown increased the mRNA expression of pS2 (Fig 2D). These results indicate that sLZIP represses the expression of estrogen-responsive genes via regulation of ERα activity.

### sLZIP physically interacts with ERα and inhibits binding of ERα to the target gene promotor

**Fig 3:**
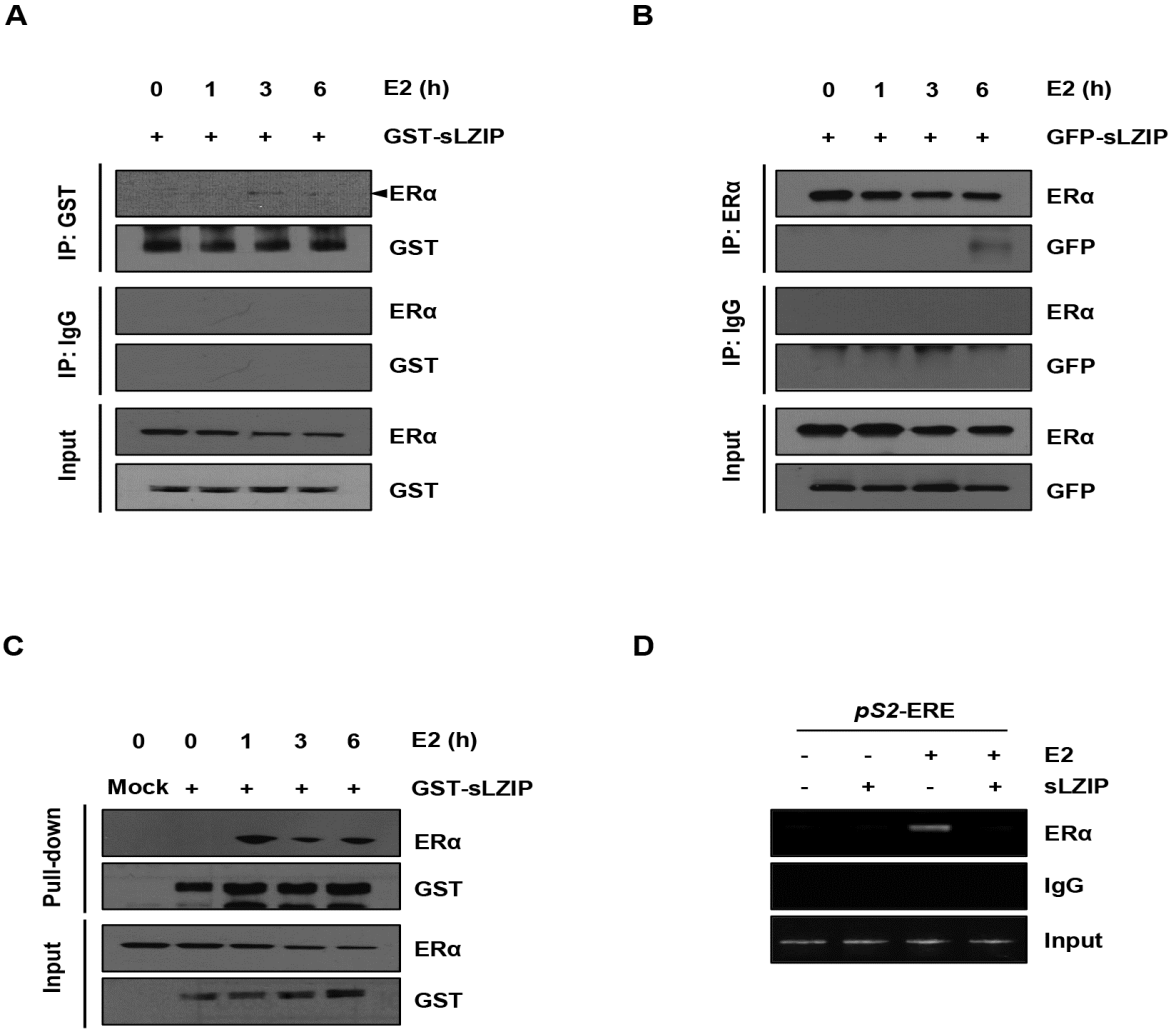
sLZIP physically interacts with ERα and inhibits binding of ERα to the target gene promotor. (A) MCF7 cells were transfected with GST-sLZIP (2 μg) and stimulated with 100 nM E2 for indicated time periods in charcoal stripped media. Total cell lysates were prepared and immunoprecipitated with IgG and anti-GST antibody. (B) MCF7 cells were transfected with GFP-sLZIP (2 μg) and stimulated with 100 nM E2 for indicated time periods in charcoal stripped media. Total cell lysates were prepared and immunoprecipitated with IgG and anti-ERα antibody. (C) MCF7 cells were transfected with GST-mock and GST-sLZIP (2 μg) and treated with 100 nM E2 for indicated time periods in charcoal stripped media. Total cell lysates were prepared, and GST pull-down assay was performed using GST beads. Physiological interaction of sLZIP with ERα was determined by Western blotting using anti-ERα and anti-GST antibodies. (D) The recruitment of ERα and sLZIP to ERα target promoters was determined by ChIP assay. MCF7 cells were transfected with GST-mock and GST-sLZIP (5 μg) and stimulated with or without 100 nM E2 for 24 h in charcoal stripped media. DNA-protein complexes were precipitated with anti-ERα antibody. Purified DNA fragments were subsequently confirmed by semi-qRT-PCR using pS2-ERE specific primers. IgG was used as a negative control.

Since the first LxxLL motif of sLZIP is required for negative regulation of ERα transcriptional activity, and LxxLL motifs bind to the nuclear receptors, we investigated whether sLZIP interacts with the endogenous ERα in breast cancer cells. MCF7 cells were transfected with GST-sLZIP and stimulated with E2 for 6 h, after which they were subjected to immunoprecipitation. ERα was co-immunoprecipitated with sLZIP at 3 to 6 h after E2 treatment (Fig 3A). We also performed immunoprecipitation using anti-ERα antibody. sLZIP was co-immunoprecipitated with ERα at 6 h after E2 treatment (Fig 3B). To confirm the interaction between ERα and sLZIP, we performed the GST pull-down assay. Results showed that ERα interacted with sLZIP at 1 to 6 h after E2 treatment (Fig 3C). These results indicate that sLZIP interacts with endogenous ERα in response to estrogen in breast cancer cells. We next examined whether sLZIP influences the recruitment of ERα to the target gene promoter using ChIP assay. sLZIP reduced the E2-dependent recruitment of ERα to ERE of the pS2 promoter (Fig 3D). These results indicate that sLZIP inhibits ERα recruitment to ERE of the ERα target genes in response to estrogen.

### sLZIP represses proliferation of breast cancer cells

**Fig 4:**
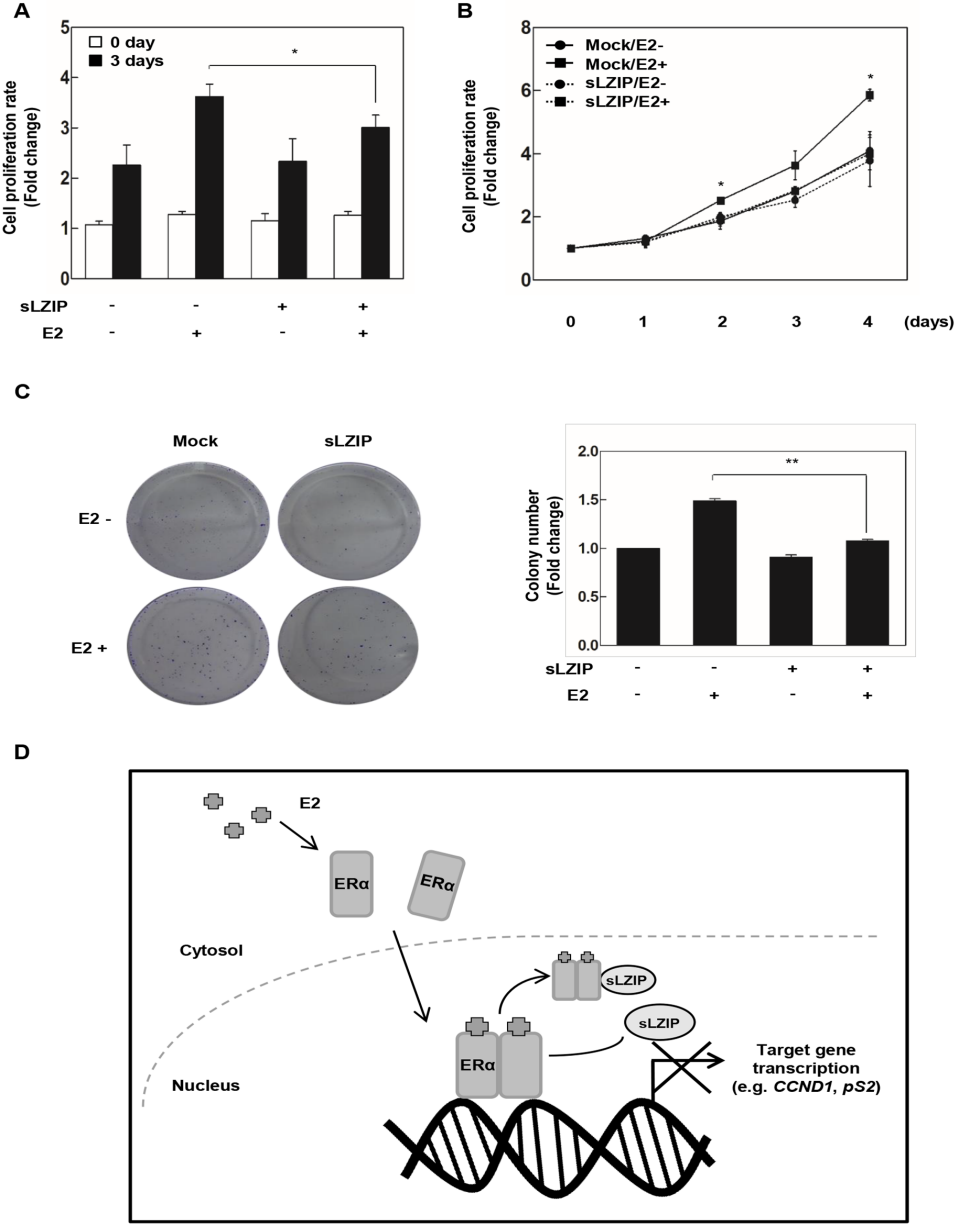
sLZIP represses proliferation of breast cancer cells. (A-B) MCF7 cells were transfected with GST-mock and GST-sLZIP (1 μg). Cells were treated with or without 100 nM E2 for indicated time periods in charcoal stripped media. Cells were treated with water-soluble tetrazolium salt solution for 30 min, and the absorbance was measured at 450 nm. (C) MCF cells were transfected with GST-mock and GST-sLZIP (1 μg). Cells were treated with or without 100 nM E2 for 6 days in charcoal stripped media. Colonies were fixed with 4% paraformaldehyde and stained with crystal violet solution. (D) Schematic diagram is based on the results of this study. Upon the ligand binding, ERα is activated and recruited to the target gene promoter, and the transcription is processed. Binding of sLZIP to these complexes inhibits recruitment of ERα to the target gene promoter, resulting in suppressing ERα target gene expression and cell proliferation. All *p* values were obtained using unpaired two-tailed *t*-test. *p < 0.05, **p < 0.01.

Since sLZIP negatively regulates ERα transactivation, we investigated the effect of sLZIP on cell proliferation in breast cancer cells. In MCF7 cells transfected with the mock vector, E2 increased the cell proliferation by 3.8-fold after 3 days of incubation, compared to a control (Fig 4A). However, cell proliferation was limited to 3.1-fold by sLZIP in the presence of E2, compared to a control (Fig 4A). We also examined the effect of sLZIP on the time-dependent proliferation of breast cancer cells. sLZIP suppressed proliferation of MCF7 cells in the presence of E2 (Fig 4B). The colony forming assay was done in order to examine the effect of sLZIP on the colony forming ability of breast cancer cells. MCF7 cells were transfected with sLZIP and treated with E2 for 6 days in charcoal stripped media. The colony number and cell density of cells transfected with sLZIP decreased approximately 30% in the presence of E2, compared with cells transfected with the mock vector (Fig 4C). These results indicate that sLZIP inhibits the proliferation of breast cancer cells via modulation of the transcriptional activity of ERα.

## Discussion

Breast cancer is typically a hormone-dependent tumor in which estrogen increases proliferation and development of the cancer [18–20]. Estrogens mediate their effects after binding to ERs, including ERα and ERβ, which are the members of the nuclear receptor superfamily that function as ligand-dependent transcription factors [21–23]. ERα interacts with a number of coactivators and corepressors [24, 25]. The transcriptional activity of ERα is enhanced by binding to coactivators, while the ERα-mediated transcription is repressed by binding to corepressor proteins [26]. Therefore, identification and characterization of cofactors of ERα are important to understand estrogen-mediated development of breast cancer.

In ERα-positive breast cancer cells, we observed a negative regulatory effect of sLZIP on the ERα transcriptional activity. sLZIP contains two LxxLL motifs at its N-terminal region, and a proline-rich C-terminal domain. Results of sLZIP mutant analysis showed that both the N-terminal and C-terminal of sLZIP are critical in ERα transcriptional repression. Therefore, the LxxLL motifs of sLZIP are probably required for association with ERα, and the C-terminal of sLZIP might contribute to the recruitment of additional cofactors. The C-terminal of sLZIP contains the proline-rich motif that can interact with other proteins. The PPLE (306–309 amino acids) motif of sLZIP can bind to WW domain-containing proteins and the PILP (324–327 amino acids) motif of sLZIP can bind to SH3 domain-containing proteins [27]. The C-terminal of sLZIP may recruit transcriptional corepressors that can suppress the transcriptional activity of ERα. The LxxLL motif (also referred to as a NR box) has been identified as a binding motif of non-DNA binding transcriptional cofactors which bind to the DNA-binding proteins, such as the nuclear hormone receptors [28]. Co-crystallization studies reveal that the LxxLL-containing peptides consist of the complexes with the ligand-binding domains of ERα and PPARγ, and that the LxxLL sequence forms an α-helix that fits into a groove on the surface of the nuclear receptor [29, 30]. It has been reported that both LxxLL motifs of LZIP could be incorporated into α-helices [15].

Our results show that overexpression of sLZIP effectively decreased the mRNA and protein levels of pS2; however, sLZIP had no effect on the ERα expression. Knockdown of endogenous sLZIP increased pS2 expression that was induced by estrogen in MCF7 cells. The physical interaction between sLZIP and ERα was examined using *in vitro* GST pull-down and immunoprecipitation assays. Results show that sLZIP directly binds to ERα upon estrogen treatment. Further studies are required for understanding the interaction mechanism between sLZIP and ERα. It includes mapping the ERα-interacting region of sLZIP, and determining which domain of ERα interacts with sLZIP. We found that sLZIP inhibits the estrogen-stimulated recruitment of ERα to ERE of the target gene promoter. We also found that cell proliferation of ERα-positive breast cancer cells is inhibited by sLZIP. These results indicate that sLZIP inhibits the ERα transactivation and estrogen-mediated ERα signaling, leading to suppression of breast cancer progression.

LZIP (also known as CREB3) is involved in diverse cellular signaling, including proliferation and migration [31, 32]. LZIP induces autophagy through the ATG5-dependent pathway [31]. LZIP increases migration by inducing CXC chemokine receptor type 4 expression in human metastatic breast cancer cells [32]. It has previously been reported that sLZIP regulates proliferation and migration [16, 33]. sLZIP plays different roles in cancer development depending on cancer type. It has been reported that sLZIP functions as a tumor suppressor in androgen-dependent prostate cancer, but sLZIP acts as a tumor promotor in androgen-independent prostate cancer [16]. In addition, sLZIP enhances migration and invasion of cervical cancer cells, leading to tumor progression [33]. sLZIP probably recruits different cofactors depending on cancer cell type and microenvironment. Further study is needed to clarify the exact roles of sLZIP in various cancers.

We are expecting that sLZIP has other unrevealed functions in cells, which may also suppresses the malignancy of breast cancer. sLZIP is a novel transcriptional corepressor of ERα, and a critical modulator of breast cancer development. Therefore, sLZIP provides a new strategy for ERα-positive breast cancer therapy.
